# Immunity revealed: Stratifying hepatocellular carcinoma for precision immunotherapy

**DOI:** 10.1016/j.omton.2025.200939

**Published:** 2025-02-04

**Authors:** Konstantinos Arvanitakis, Georgios Germanidis

**Affiliations:** 1 Division of Gastroenterology and Hepatology, First Department of Internal Medicine, AHEPA University Hospital, Aristotle University of Thessaloniki, St. Kiriakidi 1, 54636 Thessaloniki, Greece; 2 Basic and Translational Research Unit, Special Unit for Biomedical Research and Education, School of Medicine, Faculty of Health Sciences, Aristotle University of Thessaloniki, 54636 Thessaloniki, Greece

## Main text

The advent of immunotherapy has marked a paradigm shift in the treatment of hepatocellular carcinoma (HCC), a disease notorious for its high mortality rates and limited treatment responses.^1^ Despite advances in immune checkpoint inhibitors (ICIs) and combination therapies, the survival benefit remains modest, with only a subset of patients exhibiting durable responses. This underscores the critical need for biomarkers and robust classification systems to better predict therapeutic outcomes. A recent study by Liu et al. addresses this challenge by stratifying HCC patients into Immunity-High (Immunity-H) and Immunity-Low (Immunity-L) subtypes based on immune phenotypes, identifying Brother of Cell Adhesion Molecule-Related/Downregulated by Oncogenes (BOC) as a potential biomarker that predicts the response to immunotherapy.^2^ The findings of the study provide a nuanced understanding of the tumor microenvironment (TME) and its influence on therapy outcomes, proposing a framework for precision medicine in HCC. This commentary delves into the study’s key insights, situates them within the broader landscape of cancer immunotherapy, and highlights their potential impact and limitations.

The hallmark features of HCC, such as significant heterogeneity and an immunosuppressive TME, pose formidable challenges for effective treatment.^3^ The complexity of the TME, characterized by a dynamic interplay between immune and non-immune components, profoundly influences tumor progression and therapeutic efficacy.^4^ Immune cell infiltration, particularly the presence of CD8^+^ T cells, dendritic cells (DCs), and B cells, is associated with improved prognosis and better responses to ICIs. However, many patients exhibit an immunosuppressive TME, dominated by regulatory T cells, M2 macrophages, and elevated checkpoint molecule expression, leading to therapy resistance.^5^ Existing biomarkers like programmed cell death protein ligand 1 (PD-L1) expression and tumor mutation burden have shown limited predictive accuracy due to the heterogeneity of HCC. This necessitates the identification of alternative biomarkers and the development of stratification systems to guide therapeutic decisions. Liu et al. contributed significantly to this effort by leveraging transcriptomic data to classify patients into immune subtypes and identifying prognostic and predictive biomarkers for immunotherapy.^2^

Liu et al. analyzed 371 HCC samples using single-sample gene set enrichment analysis to define immune gene expression profiles, resulting in the classification of patients into Immunity-H and Immunity-L subtypes.^2^ Immunity-H patients demonstrated higher immune scores, stromal scores, and lower tumor purity, indicative of a robust anti-tumor immune response. Clinically, these patients exhibited improved progression-free survival and overall survival, supporting the prognostic value of immune profiling. Key biomarkers identified included BOC, V-set and transmembrane domain containing 1 (VSTM1), and PR domain-containing member 12 (PRDM12), with BOC emerging as a central player. Elevated BOC expression correlated with increased infiltration of CD8^+^ T cells, DCs, and B cells, as well as enhanced expression of immune checkpoint molecules like PD-L1 and CTLA4. Functionally, BOC overexpression *in vitro* reduced tumor cell proliferation and invasiveness, aligning with its role as a positive prognostic factor. Importantly, high BOC expression predicted favorable responses to anti-PD-1 therapy, highlighting its potential as a biomarker for selecting patients likely to benefit from ICIs.

The study’s stratification framework has broad implications for HCC treatment and cancer immunotherapy at large. By integrating genomic, transcriptomic, and immune cell infiltration data, it provides a holistic approach to understanding the TME and tailoring therapeutic strategies. The identification of BOC as a biomarker bridges a critical gap in patient stratification, enabling more precise predictions of immunotherapy efficacy. These findings also underscore the importance of considering the heterogeneity of the TME in therapeutic decision-making. For example, Immunity-H patients, characterized by enriched immune activation pathways such as interferon-γ signaling, may benefit from ICIs or combination therapies that amplify immune responses. Conversely, Immunity-L patients may require alternative strategies, such as TME modulation or therapies targeting immunosuppressive cells. In addition to clinical stratification, the findings contribute to our understanding of how specific genes like BOC influence the TME. By modulating immune cell infiltration and checkpoint molecule expression, BOC appears to shape the immune landscape in ways that enhance therapeutic responsiveness. Such insights are invaluable for designing targeted therapies that can selectively exploit these immune dynamics, potentially improving treatment outcomes for a broader range of patients.

While the study offers valuable insights, several limitations warrant discussion. The retrospective nature of the analysis and reliance on public datasets may introduce biases. Prospective validation in larger, more diverse cohorts is essential to confirm the findings and establish clinical utility. Additionally, the study’s focus on transcriptomic data, while informative, may not fully capture the dynamic interactions within the TME. Emerging technologies like single-cell RNA sequencing and spatial transcriptomics could provide a more granular view of immune cell distribution and function. Moreover, the functional role of BOC in modulating the TME and its interactions with other pathways also require further investigation. As a component of the Hedgehog signaling pathway, the involvement of BOC in immune regulation and tumor biology could reveal new therapeutic targets, while exploring the interplay between BOC and other biomarkers such as VSTM1 and PRDM12 may offer additional insights into the immunogenomic landscape of HCC. Understanding the mechanisms by which these biomarkers interact with systemic therapies could also inform combination treatment strategies. For instance, combining ICIs with agents that enhance BOC expression or activity might yield synergistic effects, further improving therapeutic outcomes. Such combinations could be particularly beneficial for patients with intermediate immune profiles who do not clearly align with Immunity-H or Immunity-L classifications.

The findings of Liu et al. represent a step forward in the quest for precision immunotherapy in HCC.^2^ In the coming years, we anticipate significant advancements driven by multi-omics approaches, integrating genomics, proteomics, and metabolomics to refine immune classification systems. The incorporation of artificial intelligence and machine learning could further enhance biomarker discovery and predictive modeling. Combination therapies that synergize ICIs with agents targeting the TME, such as angiogenesis inhibitors or metabolic modulators, are likely to gain prominence. The stratification framework proposed in the Liu et al. study could guide the design of such combination regimens, optimizing therapeutic outcomes for distinct patient subgroups. Finally, the integration of immune profiling into clinical workflows will require collaboration among researchers, clinicians, and policymakers. Standardizing methodologies and ensuring equitable access to advanced diagnostic tools will be critical to translating these findings into real-world benefits.

In conclusion, the study by Liu et al. exemplifies the power of immunogenomic profiling to unravel the complexities of HCC and guide therapeutic innovation.^2^ Stratifying patients based on immune phenotypes and identifying biomarkers like BOC lays the groundwork for more personalized and effective immunotherapy strategies. As the field evolves, integrating these insights with emerging technologies and therapeutic modalities promises to transform the landscape of HCC treatment, offering hope to patients battling this formidable disease.

## Author contributions

Conceptualization, K.A. and G.G. Writing – original draft, K.A. Writing – review & editing, G.G. Supervision, G.G.

## Declaration of interests

The authors declare no competing interests.
